# Asynchronous Stepped Fourier Transform Ion Mobility Spectrometry

**DOI:** 10.1021/jasms.5c00220

**Published:** 2025-12-08

**Authors:** Emily Edstrom, Saned Gharari, Eric Davis

**Affiliations:** Whitworth University, Spokane, Washington 99251, United States

## Abstract

Fourier Transform is a low-cost method for improving duty cycle, resolving power, and signal-to-noise ratio in the Ion Mobility Spectrometry (IMS) experiment in a 2-gate IMS cell. By simultaneously pulsing both gates through a frequency sweep, the resulting data may be deconvoluted into a time-based mobility spectrum through a Fast Fourier Transform (FT). However, inconsistencies common in low-cost function generators result in spectral artifacts. In this work, an asynchronous stepped frequency FTIMS method is demonstrated, which uses a simple, software timed pulse generator compatible with any modern Analog-Digital Converter (ADC) system. By unlinking the frequency initiation and data collection using a long rise-time amplifier circuit, a stand-alone FTIMS with Faraday Plate detection was characterized in both single gate and FT modes of operation using the same IMS cell. Asynchronous stepped FTIMS parameters were investigated for system optimization with respect to resolving power, signal-to-noise ratio, and experimental time. Once optimized, asynchronous FTIMS demonstrated significant improvements in resolving power and signal-to-noise ratios without a significant increase in experimental time. By unlinking the frequency generation and data analysis, a simple Python script was demonstrated using a variety of commercially available ADC systems ranging in cost from several thousand to several hundred dollars (USD) without sacrificing spectral fidelity. A custom circuit was developed to allow a Raspberry Pi 4 Single Board Computer (SBC) to function as the data acquisition and control (DAC) interface for a low-cost stand-alone FTIMS solution.

## Graphical abstract

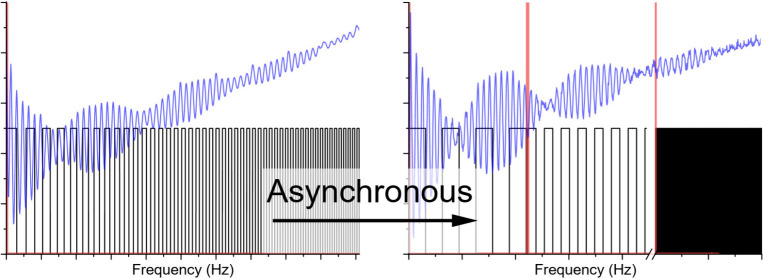

## Introduction

As a mature technology, Ion Mobility Spectrometry (IMS) is utilized globally in security, military, and scientific applications.^1–5^ Its ease of use and ability to separate gas phase ions on the basis of size-to-charge ratios is complementary of Mass Spectral (MS) mass/charge separations and is frequently coupled to MS measurements to provide 2D data. IMS has numerous examples of increasing data available through other analytical methods such as MS,^6^ GC,^7,8^ or HPLC.^9^ The utilization of IMS on a broader scale is limited by the instrumental requirements of this technique, including high frequency pulse generation, amplification of high speed, nA-level signals, and high-speed analog to digital conversion. Historically, these limitations have been overcome through application of commercially available amplifiers and high-speed Analog to Digital Converter (ADC) modules that come with a steep price and necessitate significant instrumental and programming experience to implement the IMS experiment.

More recently, commercially available, stand-alone, research-grade IMS units have entered the market, but these are severely limited in resolution and modularity due to their compact size and the nature of commercial instrumentation.^10^ In an attempt to cover this gap, Reinecke and Clowers developed an open-source IMS platform in 2018 where they described a PCB-based IMS cell and released open source amplifiers.^11^ Recent literature has also demonstrated inexpensive high voltage power supplies^12^ and electrostatic gate drivers.^13^ However, the use of PCB-based designs, while simple and inexpensive, preclude the ability to heat the IMS cell. While this limitation is insignificant for many IMS applications using radioactive or corona-based ionization methods, ElectroSpray Ionization (ESI) generated ions have proven difficult at ambient temperatures due to desolvation kinetics.

Recently, flexible, rolled IMS cells have been developed that allow increased temperature up to 110 °C^14,15^ and have been demonstrated to produce high-resolution results.^16^ However, these flexible cells are more difficult and more expensive to construct than the stacked-ring PCB designs described by Reinecke and Clowers, and have recently been shown to produce slightly lower resolving powers when directly compared.^17^ Therefore, a need exists to produce ESI-based measurements in PCB-based instrumentation that avoids the desolvation limitations of the ESI process at ambient temperatures. This is especially important for analytes which require increased water content in the ESI solvent for analyte solvation.

Fourier Transform IMS (FTIMS) was originally developed to increase the duty cycle of the IMS experiment to 50% (as opposed to 0.1–0.5% typical in pulsed DT-IMS), thereby decreasing detection limits while simultaneously decreasing the necessity of high speed ADC capabilities.^18^ As FT methods for IMS developed, it was noted that the Fourier Transform experiment, when applied to IMS, resulted in deconvoluted spectra that ignored transient ion species in the final spectra.^19,20^ FT (and similar multiplexed methods such as Hadamard Transform) has since been utilized extensively to couple the IMS experiment to slower MS-based methods while simultaneously improving measured Signal to Noise ratio (S/N) and resolving power.^21–24^

A key metric in producing an FT-based IMS measurement is the development of the frequency sweep used on a pair of ions gates in the IMS cell. Traditionally, this is achieved through a function generator and is typically triggered to start simultaneously with the initiation of data collection through the ADC (Figure 1 – solid lines). The resultant time-domain signal is then deconvoluted through FT to produce an IMS spectrum.^25^ The exact parameters of a frequency sweep have been shown to influence the quality of data in the multiplexed method. Additionally, the quality of the function generator has been shown to significantly influence the resultant spectra. Low-cost function generators tend to produce overtones and errant peaks in the deconvoluted data. These limitations were recently overcome through the production of the frequency sweep at a binary level on an Arduino-based function generator developed for interfacing the IMS experiment with an LTQ-based mass spectrometer.^26,27^ More recently, Cabrera et al. demonstrated a nonlinear frequency modulation in FTIMS-Mass Spectrometry that improved S/N by avoiding gating inefficiencies, especially in the high-frequency regime of an FTIMS experiment.^28^ Furthering this work, the frequency sweep was coupled to a mass-spectral analysis and triggered simultaneously to the MS experiment at each desired frequency, resulting in a stepped frequency modulation that allowed exact determination of ion swarm timing and coupling of the IMS data to the MS data, and allowed signal averaging at each frequency; a method that was previously impossible with swept frequencies.^29^

**Figure 1:**
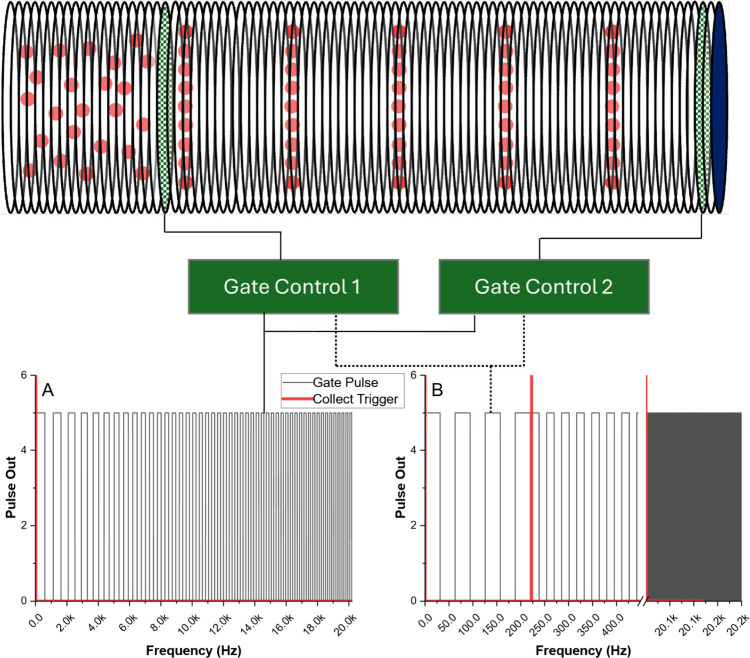
Experimental diagram for Swept Frequency FTIMS (left, solid lines) compared with asynchronous stepped FTIMS (right, dashed lines). Both gate controls were triggered simultaneously from the DAC through a BNC splitter. Gate 2 could be bypassed with a jumper for single gate averaged signal operation and comparison. Lower figures demonstrate the swept vs stepped pulsing diagram. Note repeated pulses at each step frequency in the stepped method of operation and continuous synchronization between data collection and frequency step on the collection triggers in the stepped method.

The work herein describes the application of a stepped frequency modulation method on a stand-alone, ambient temperature, stacked PCB IMS cell using electrospray ionization. Key to this work is the demonstration that precise control and triggering of data acquisition with respect to frequency pulse is unnecessary due to the long amplifier rise times used in stand-alone FTIMS experiments. This allows the utilization of software timing, significantly decreasing the cost of DAC hardware. When coupled with the recently described amplifiers and control hardware, this work demonstrates stepped FTIMS utilizing high speed National Instruments (NI) hardware, inexpensive Digilent Analog Discovery hardware, and a complete Raspberry Pi control system using inexpensive frequency generators and DAC HATs.^30^ Through the use of the Python programming language, the code for any of these solutions is similar and deployable to a large number of DAC solutions with minimal code modifications.

## Experimental Section

### IMS System

The IMS cell used was a stacked PCB design as described previously.^11^ The cell consisted of 15 rings in the reaction region and 47 in the drift region separated by a 3-grid ion gate using a FET pulser previously described.^13^ A second ion gate was positioned between the final ring and the Faraday plate detector for the FTIMS experiments. The central grid was wired to match the electric field in this region for single gate experiments in order to compare results using the same IMS cell. The electric field was held constant at 350 V/cm and ionization was achieved through orthogonal corona discharge and electrospray ionization held at a 2,500 V bias to the first ring of the IMS cell. High voltage was produced through supplies described previously.^12^

### Reagents and Samples

Corona Discharge Ionization (CDI) was used for all Reactant Ion Peak (RIP) experiments, as well as for di-*tert*-butylpyridine (DtBP) and dimethylmethylphosphate (DMMP), which were introduced as headspace vapor immediately prior to the first ion gate. CDI was accomplished using a sharpened needle placed orthogonal to the entrance screen and biased to ∼ 2,500 V above the front ring.^32^ For experiments requiring liquid samples, sample introduction was accomplished through glass capillary electrospray ionization^31^ using 50/50 methanol/water as the solvent at a flow rate of 4 μL/min positioned orthogonal to the front screen of the IMS cell. Reagents were obtained as either neat samples (Tetraalkylammonium bromide salts – tetrapropylammonium (T3A), tetrabutylammonium (T4A), tetrapentylammonium (T5A) and tetrahexylammonium (T6A)) or as 1000 ppm standards in acetonitrile (Cocaine, nitroglycerine (NG), and 2,4,6-trinitrotoluene (TNT)) and diluted to 10 ppm in ESI solvent prior to sample introduction. All reagents were purchased in analytical standard grade from Millipore-Sigma (St. Louis, MO).

### Stepped Frequency Experiments

A Python script (provided in Supporting Information) was created and modified for each DAQ device described herein. Contrary to prior work,^29^ synchronization was not required in these experiments due to the long rise time used on the amplifier system. A 2-gate IMS cell with matched pulse frequencies produces a steady-state DC signal dependent on the mobilities of the ions in the swarm, so direct synchronization of the gate pulses to the data collection were found to have negligible effect on the overall signal. This also precludes the necessity of high-accuracy hardware-timed signal measurement, so an average over time was taken at each frequency. Therefore, this work is a combination of the stepped frequency method with the swept frequency methods previously demonstrated, allowing the system to be operated without the precision binary controller described by Cabrera et al.^29^ Frequencies were stepped from a user-selectable minimum to maximum values with a selectable frequency step and number of averages. Averages were obtained for a set period of time, not the number of pulsed frequencies, and were developed based on the slowest frequency measured. For example, a 4-average measurement with the minimum frequency of 4 Hz stepped by 5 Hz to a maximum frequency of 4000 Hz would result in summing the overall signal obtained over 1 s at each desired frequency step (Figure 1, lower-right; note multiple pulses per step at each frequency). This time period was held constant for all observed frequencies, dependent only on the starting frequency used. A Keithley (Cleveland, OH) 427 Current–Voltage amplifier set to 10^9^ V/A and a 100 ms rise time was used for raw signal amplification for all FT experiments. A 0.1 ms rise time was used for all SG IMS experiments. For pulse generation and data collection, a National Instruments (Austin, TX) USB-6341 multifunction DAQ, a Digilent (Pullman, WA) Analog Discovery 2, Analog Discovery 3, and a Raspberry Pi 4 with a Digilent MCC118 DAQHAT and custom pulsing circuit based on a DDS AD9850 signal generator module (HiLetgo, Shenzhen, China) circuit (circuit provided in Supporting Information) were used for pulse generation and data collection. Python scripts were modified to allow this data acquisition to occur using all DAC systems demonstrated. Gate pulsing was accomplished using a pair of gate pulsers previously described^13^ with the output from the DAC device split to both controllers simultaneously (Figure 1 – dashed lines connected to gate controllers). Data acquisition was software triggered 0.5 ms after establishing the new frequency (Figure 1 – Red pulses).

### Single-Gate Experiments

The PCB IMS utilized in this work was designed to function in both single gate (SG) and dual gate (FT) modes with minimal modifications to the experiment. When operated in SG mode, the second gate was jumpered into the open position without removing the gating grids in order to maintain an identical cell length and geometry for direct comparison. In this way, both resolving power and S/N could be directly compared between the SG and FT experiments. All SG experiments used a National Instruments USB-6341 DAQ for gate pulse and data acquisition control. All pulsing and data acquisition was controlled with an in-house Python script (available in Supporting Information) using a 50 ms scan time, 0.2 ms gate pulse width, and 275 averages.

### Spectral Comparisons

All spectra demonstrated were analyzed by resolving power [eq 1] following peak fitting in OriginLab 2024 (Northampton, MA).

1 $$R_{p}=\frac{t_{d}}{w_{1/2}}$$

Where *t*_d_ is the observed drift time (peak center) of the peak and $w_{1/2}$ is the Full Width Half-Maximum (fwhm) of the observed peak. Signal-to-noise ratio (S/N) was measured assuming maximum peak height following peak fitting in OriginLab and using RMS noise of the observed spectrum [2]:

2 $$\mu_{\mathrm{RMS}}=\sqrt{\frac{\underset{i}{\sum }{\left(H_{i}-\mathrm{H̅}\right)}^{2}}{n}}$$

## Results and Discussion

In comparison with a swept frequency FTIMS experiment (4–8000 Hz over 8 s, 100 averages, obtained with an Analog Discovery 2 function generator), Figure 2 correlates with Figure 1, showing the similarities between the operational modes swept (A) and stepped (B) frequency experiments in the time (top) and mobility (bottom) domains. The stepped frequency, as previously reported, avoids artifacts (small spikes at regular intervals in the swept frequency-domain signal) inherent in many function generators and provides a higher S/N spectrum in a similar time period and simpler experiment.

**Figure 2:**
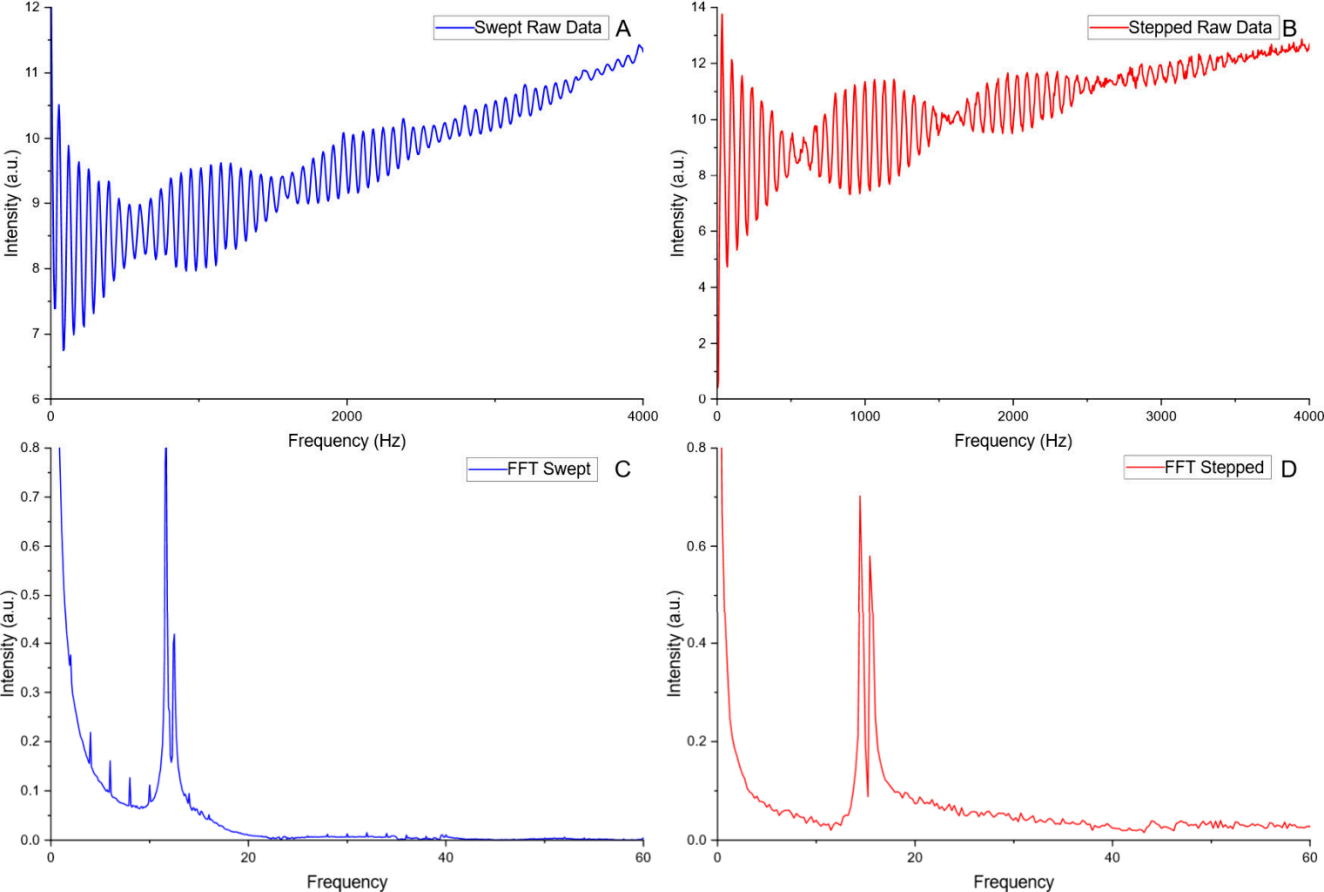
Swept (A, C) vs stepped (B, D) FTIMS time-domain and mobility-domain spectra demonstrating the similarities between the methods of operation. Note the artifacts present in the swept spectrum are absent in the stepped-mode signals. Resolving power and signal-to-noise ratio are similar.

To determine the optimal final frequency for the stepped FTIMS experiment, four ending frequencies in a range from 1 to 8 kHz were tested for resolving power and the average time required for experimental completion (Figure 3). An initial frequency of 2 Hz was held and stepped by 2 Hz until reaching a final frequency between 1 and 8 kHz. Increasing the final frequency produced spectra with sharper peaks and increased resolving power, but decreased signal intensity, dropping by 95% as the ending frequency increased from 1 to 8 kHz. However, a lower ending frequency was correlated to a decreased observed resolving power, which was especially noted from 1 to 4 kHz where the resolving power increased by 400%. This trend was demonstrated as statistically insignificant (p = 0.165) between 4 and 8 kHz, while 8 kHz required double the experimental period with no observed increase in spectral quality. For example, a starting frequency of 2 Hz with a 2 Hz step up to a maximum of 4 kHz required approximately 8 min, while ending at 8 kHz required 16 min. Therefore, a final frequency of 4 kHz was used in all subsequent experiments for optimized experimental period and resolving power.

**Figure 3:**
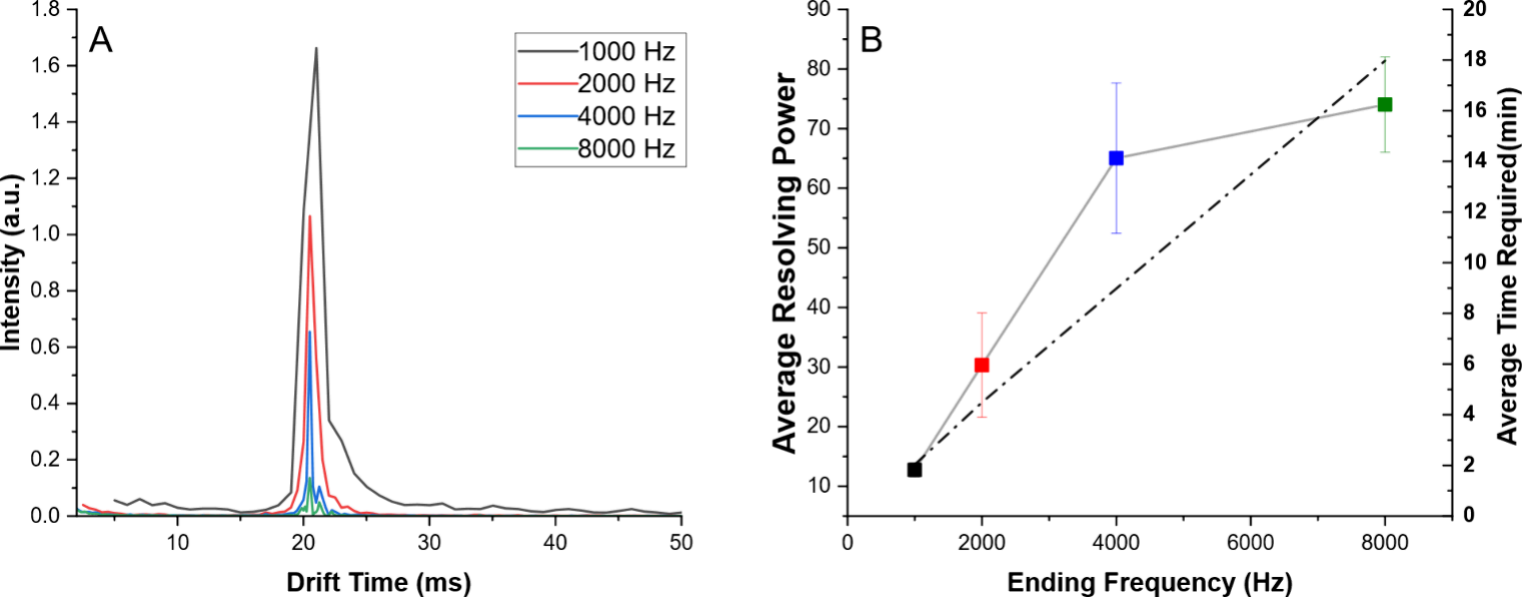
Optimization of final frequency measured in Fourier Transform IMS. Each experiment was held at the same initial frequency of 2 Hz and used a frequency step of 2 Hz as an ideal use case. (A) Overlay of average signals after Fourier Transform. Increased final frequency resulted in sharper peaks and a higher resolving power at the cost of overall signal. (B) Comparison of resolving power (points, solid line) for each final frequency and average experimental time required (dashed line). A significant increase in resolving power was noted from 1 to 4 kHz final frequency, with a statistically insignificant increase from 4 to 8 kHz. Increasing the final frequency from 4 to 8 kHz doubled the experimental time required and resulted in a significant loss in overall signal-to-noise, so all subsequent experiments used 4 kHz as the final frequency in a compromise between time required, signal, and resolution. Error bars indicate 1 standard deviation from the mean across triplicate measurements.

While the final frequency was held at 4 kHz (Figure 3), the initial frequency was optimized with a 2 Hz step across a range of 2–10 Hz (Figure 4). Neither the signal intensity nor observed resolving power demonstrated any trend as evidenced in Figure 3. The highest mobility peak (reactant ion peak) was observed with a drift time of approximately 20 ms, corresponding to a frequency of 50 Hz. Therefore, it is hypothesized the initial frequencies in this work (2–10 Hz) provided sufficient separation from the observed ion frequency to maintain spectral accuracy in the time-domain signals. Significantly faster initial frequencies would likely have an impact on the observed spectrum, so a 10 Hz frequency was used throughout the subsequent experiments to minimize experimental time without affecting resolution.

**Figure 4:**
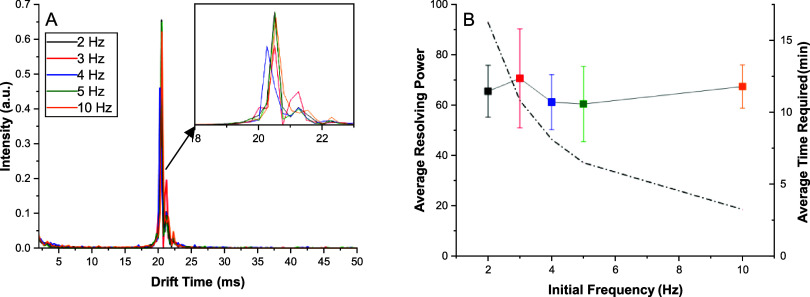
Optimization of starting frequency measured in FTIMS. For consistency, each experiment used a 4000 Hz final frequency as optimized in Figure 2 and used a 2 Hz frequency step. (A) Overlay of experimental signals with a close-up comparison of the peaks measured, demonstrating no significant difference between the measured signals regardless of the initial frequency used. (B) Comparison of resolving power (points, solid line) for each initial frequency and the average experimental time required (dashed line). Each data point was obtained at the time required for the lowest frequency (e.g., 2 Hz with 2 averages used a period of 1 s over which the signal was averaged). Thus, initial frequency had a significant effect on the experimental time. Error bars indicate 1 standard deviation from the mean across triplicate measurements.

The frequency step served as the final variable for consideration in the asynchronous stepped FTIMS experiment. While measuring experimental time and resolving power, as in Figures 3 and 4, the frequency step was varied over a range from 1 to 5 Hz (Figure 5). An initial frequency of 10 Hz and a final frequency of 4000 Hz were used, as previously determined (Figures 3 and 4). Similar to the trend observed for the initial frequency, the frequency step had no significant effect on the intensity or resolving power of the observed signal. Additionally, the frequency step used directly correlated to the experimental time required. Experiments with lower frequency steps took longer on average due to the increase in data points obtained. For example, an experiment utilizing a step of 1 Hz took almost 20 min, whereas a frequency step of 5 Hz took fewer data points and had an experimental time of less than 3 min. To optimize resolving power and experimental time, a frequency step of 5 Hz was used in all further experiments. Overall, it was found that an initial frequency of 10 Hz, a step of 5 Hz, and a final frequency of 4 kHz produced reproducible spectra with a significant increase in resolving power over a similar time period to signal averaged SG IMS experiments.

**Figure 5:**
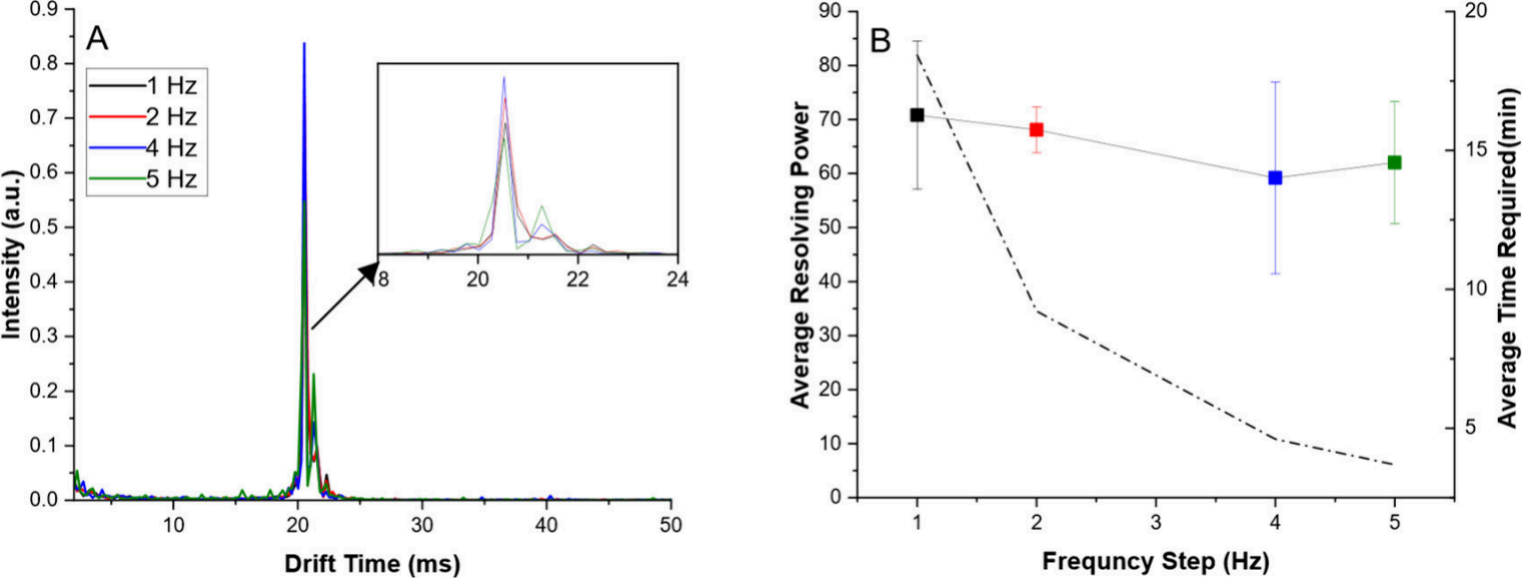
Optimization of frequency step measured in FTIMS. For consistency, each experiment used a 4000 Hz final frequency and a 2 Hz starting frequency in the data presented. (A) Overlay of experimental signals with a close-in comparison of the peaks measured demonstrating no significant difference between the measured signals regardless of the frequency step used. (B) Comparison of resolving power (points, solid line) for each frequency step and the average experimental time required (dashed line). Since decreasing frequency step increased the number of data points obtained, frequency step also had a significant effect on the experimental time required but demonstrated little change in the data obtained. All subsequent experiments used a 5 Hz frequency step. Error bars indicate 1 standard deviation from the mean across triplicate measurements.

Asynchronous Stepped Fourier Transform IMS demonstrated significantly improved resolving powers over that of traditionally single-gated IMS for a range of compounds tested (Figure 6B). IMS standards (DtBP, TXA Salts) and common IMS analytes (DMMP, Cocaine, Nitroglycerin (NG), and 2,4,6-Trinitrotoluene (TNT)) were analyzed by SGIMS and asynchronous FTIMS (example spectra in Figure 6A, C, D, and F). On average, FTIMS produced a 21% increase in resolving power across all compounds tested. This increase was statistically significant in a paired *t* test (p = 0.012). Some compounds provided significantly higher improvements over this average; for example, cocaine had a resolving power of 55 in SGIMS, and 80 in asynchronous FTIMS, a 45% increase. As another example of the utility of the asynchronous stepped FTIMS experiment, the observed spectra for TNT (Figure 6F) exhibited near baseline separation in the FTIMS experiment for all peaks, including the RIP in negative mode, a feat impossible with the same tube in the same conditions in SGIMS mode.

**Figure 6:**
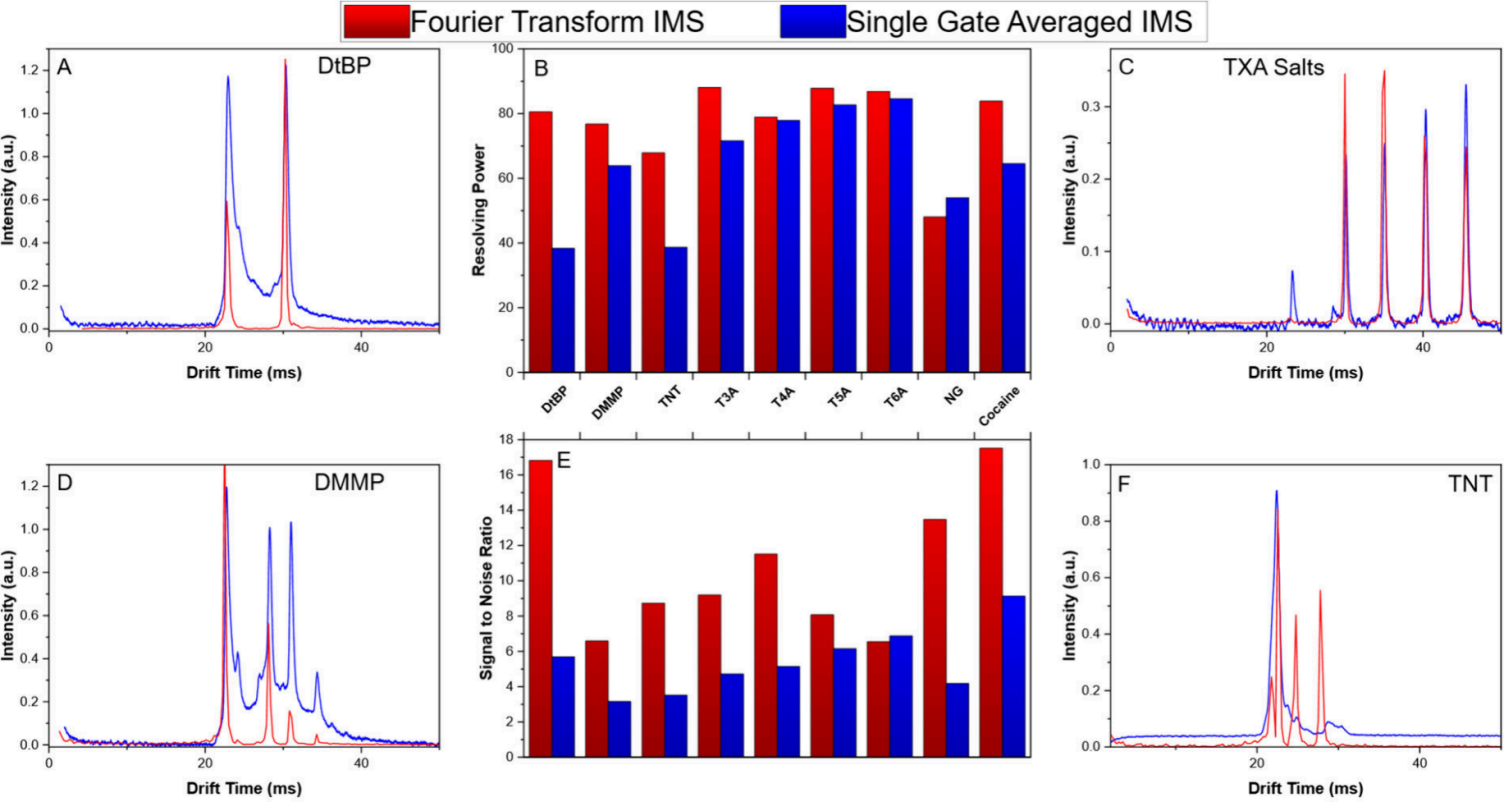
Comparison of common IMS standards and analytes with respect to resolving power (B) and signal-to-noise ratio (calculated as maximum peak height/RMS noise) (E). Representative spectra for di-*tert*-Butylpyridine (DtBP) (A), a mixture of 4 tetraalkylammonium (TXA) salts (tetrapropyl (T3A), tetrabutyl (T4A), tetrapentyl (T5A), and tetrahexyl (T6A)) (C), dimethyl methylphosphonate (DMMP) (D), and TNT (F). Asynchronous stepped FTIMS demonstrates significant improvements in both signal-to-noise and resolving power, able to baseline separate negative mode RIP, as well as providing mobility accuracy across a range of compounds. It exhibits significant reduction in transient ions observed, especially noted in DtBP (A) and DMMP (D).

In addition to the observed improvement in resolving power, asynchronous FTIMS also demonstrated significant improvement in S/N ratios for all compounds observed (Figure 6E). Similar to resolving power, the average S/N for SGIMS was 4.3 and FTIMS was 10.2, a 137% improvement. This is visibly observed in the red (FTIMS) spectra in Figure 6. For example, both cocaine and DtBP demonstrated a 100% improvement in S/N ratio compared to SGIMS. These improvements to both resolving power and S/N coincided with the expected tendency of the FTIMS experiment to reduce the presence of transient species in the IMS experiment, commonly observed as “bridging” between peaks.^33^ A result of reducing these bridged peaks, however, is a change in relative ion abundance between reactant ion and product ion peaks. This is especially evident in the spectra of both DtBP (between the reactant ion peak (RIP) and monomer ion) and DMMP (between the observed monomer and dimer peaks and between the RIP and monomer) where the RIP is sharper and shows lower total ion abundance. Removing these transient species reduced the total ion abundance (integrated peak area) while increasing resolving power. (Figure 6 – A, D). Finally, in the observed spectra, the asynchronous experiment had no effect on the observed mobility accuracy for all compounds tested. All data are numerically summarized in Table 1.

**Table 1:** Data Summary of Single Gate vs Stepped FTIMS Results in Figure 6 ^a^

	Single Gate IMS				Stepped FTIMS				
	Drift Time (ms)	Reduced Mobility (*K*_0_)	Resolving Power	S/N	Drift Time (ms)	Reduced Mobility (K_0_)	Resolving Power	S/N	Literature *K*_0_
DtBP	30.34	1.42	38.35	5.69	30.27	1.41	80.55	16.82	1.42 ^34^
DMMP (Dimer)	30.52	1.41	63.91	3.16	31.00	1.39	76.80	6.60	1.40 ^35^
Cocaine	36.71	1.16	64.55	9.13	36.67	1.16	83.90	17.52	1.16 ^36^
T3A	30.11	1.43	71.62	4.72	30.00	1.44	88.12	9.20	1.51 ^31^
T4A	35.06	1.23	77.89	5.14	35.09	1.23	78.96	11.51	1.28 ^31^
T5A	40.33	1.07	82.73	6.16	40.18	1.07	87.86	8.08	1.10 ^31^
T6A	45.49	0.95	84.61	6.88	45.53	0.95	86.85	6.55	0.97 ^31^
NG	24.93	1.71	54.00	4.18	24.70	1.72	48.07	13.47	1.83 ^1^
TNT	28.78	1.48	38.71	3.52	28.42	1.50	67.92	8.73	1.54 ^37^

a Literature mobility values provide references for each observed species for comparison.

As the asynchronous stepped FTIMS experiment greatly simplifies the timing requirements over previously published results, a range of data collection methods and digital-analog converters (DAC) may be utilized. For all experiments in Figures 3–6, a National Instruments USB-6351 provided gate pulse timing and DAC. However, Figure 7A demonstrates a RIP spectrum using the NI USB-6351, a Digilent Analog Discovery 2, Digilent Analog Discovery 3, and a custom Raspberry Pi circuit combined with a Digilent DAQHat MCC118 for DAC (BOM and circuit design provided in Supporting Information). The resolving power was compared for resultant RIP in FTIMS mode (Figure 7B). The RIP of the NI USB-6351, the most expensive DAC, had the highest resolving power and most consistent results of all DAC methods, but did not show a statistical difference from the AD3 or Raspberry Pi-based systems. The efficacy of the Raspberry Pi DAC method in conjunction with the PCB IMS cell demonstrates that the asynchronous stepped FTIMS experiment can be conducted in a low-cost system, increasing accessibility for implementation of this technology.

**Figure 7:**
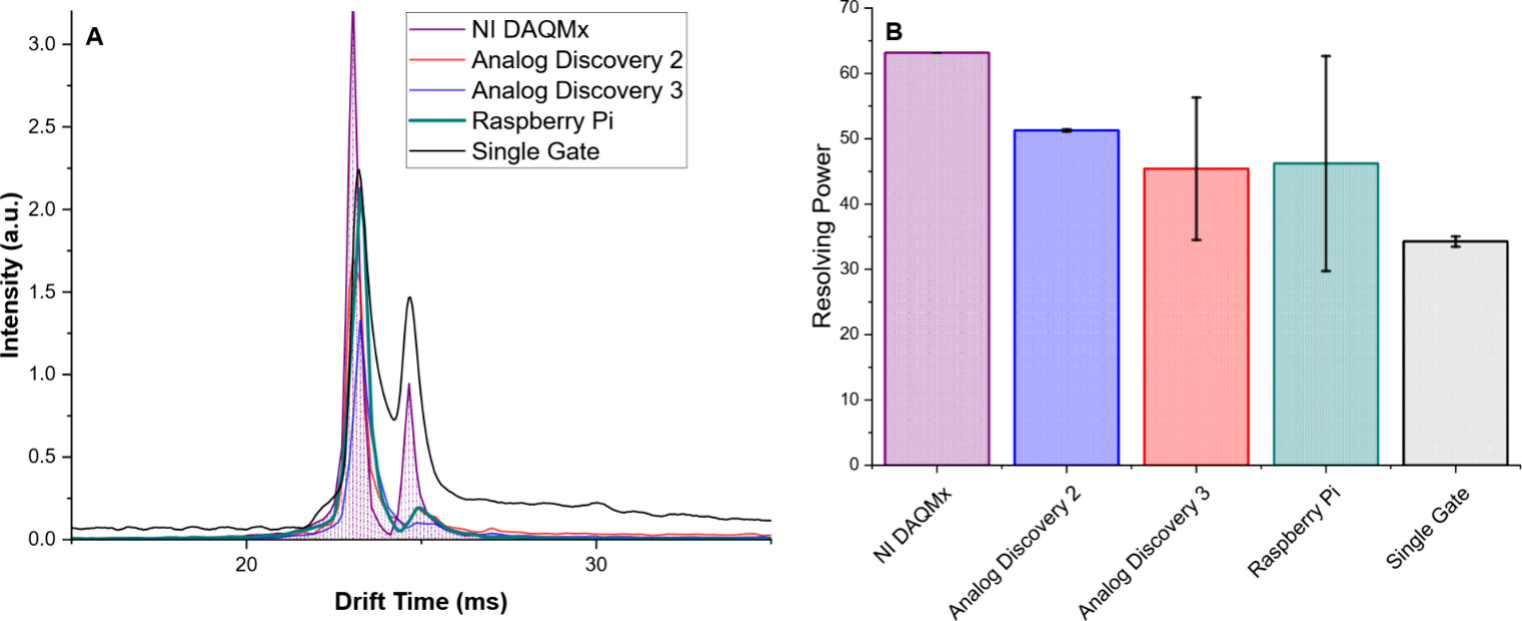
Comparison of data collection methods in FTIMS mode of RIP. (A) Overlay of all DAC methods used and a single-gate spectrum (black). Filled in purple trace used the same NI DAC as all other data demonstrated herein. The Analog Discovery series by Digilent produced similar results to the Raspberry Pi based DAC with respect to the measured resolving power of the first RIP peak (B). Error bars indicate 1 standard deviation from the mean across triplicate measurements. While the NI DAC produced superior results with respect to both resolving power and standard deviation, it represents an order of magnitude increase in experimental cost for an incremental improvement.

As prior work^25,29^ has already shown, the stepped FTIMS experiment may be implemented in an IMS-Mass Spectrometry (IMS-MS) methodology. The FTIMS experiment provided a method for slowing down the IMS experiment, but the requirement of a synchronized pulse frequency required highly customized electronics for precision timing and pulsing between the instruments. As shown in this work, removing the requirement for synchronous pulse timing will allow for this method to be utilized with any mass analyzer with minimal modifications to the MS experiment.

## Conclusions

Asynchronous Stepped FTIMS is demonstrated herein as a cost-effective method for operating a drift-tube IMS system. FTIMS has known advantages over SGIMS in terms of transient species, spectral clarity, signal-to-noise, and resolving power but requires sophisticated, high-speed DAC systems in order to produce a frequency sweep or synchronous stepped frequencies on a dual gate IMS cell. In this work, the application of an asynchronous pulse frequency to the FTIMS experiment increased both resolving power (average 45% increase) and S/N ratio (average 137% increase) along with the expected improvements in transient species and spectral clarity without the necessity for a high-precision DAC. Therefore, the implementation of asynchronous timing to the stepped FTIMS experiment significantly improved the accessibility of IMS technology while simultaneously maintaining the advantages of the FT experiment and allowing the use of inexpensive DAC solutions.

## Supplementary Material



## Notes

Python scripts for asynchronous FTIMS using NiDAQmx or Digilent (Analog Discovery 2 or 3) hardware and a script to deconvolute resultant time-domain signals and combine multiple spectra into a single file for analysis, Bill of materials and schematic diagram for Raspberry Pi interface circuit as well as a Python script for asynchronous FTIMS using this circuit, and Python scripts for single-gate IMS control with NiDAQmx hardware and combining single gate spectra to a single file for analysis (ZIP)
