# Supplementary figures and images for: Asynchronous Stepped Fourier Transform Ion Mobility Spectrometry
Source: J Am Soc Mass Spectrom. 2025 Dec 8;37(1):86–94. doi: 10.1021/jasms.5c00220 (PMC12784390; doi:10.1021/jasms.5c00220)

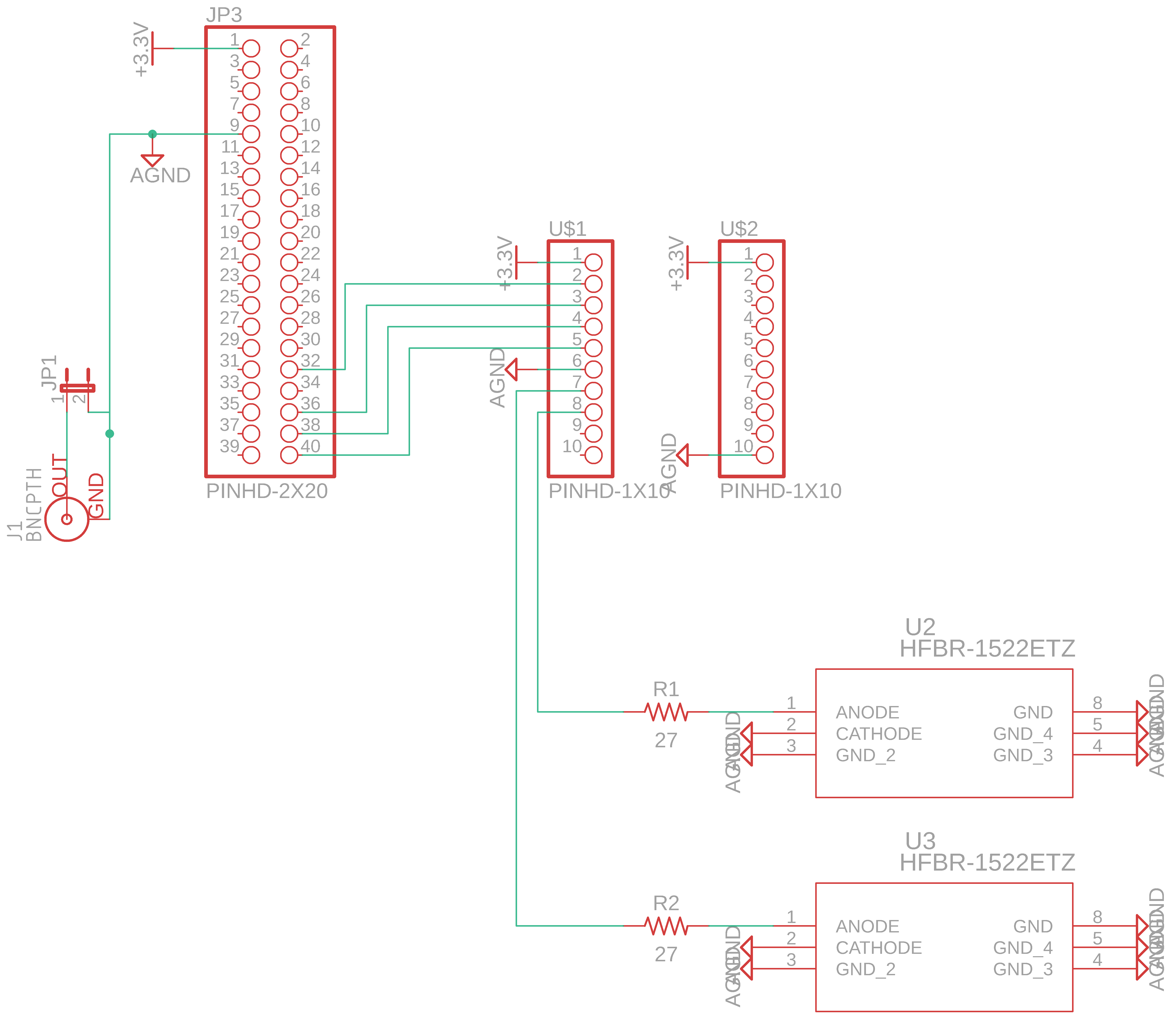


*Raspberry Pi Interface Circuit*

Supplement: Supplementary file 1 [file js5c00220_si_001.zip › Supplemental Materials/Raspberry Pi/Raspberry Pi Interface Circuit.docx]
